# The role of clinical laboratory parameters in diabetic neuropathy: correlations and recent advances

**DOI:** 10.3389/fendo.2026.1743898

**Published:** 2026-02-27

**Authors:** Ruitong Guo, Pingping Lou, Min Li, Meimei Tian, Lu Gao, Huijie Ma, Yan Liu, Xinli Jiang

**Affiliations:** 1 Department of Endocrinology, Hebei Medical University Third Hospital, Shijiazhuang, China; 2 Department of Physiology, School of Basic Medicine, Hebei Medical University, Shijiazhuang, China; 3 Department of Ophthalmology, Hebei Medical University Third Hospital, Shijiazhuang, China

**Keywords:** biomarkers, clinical laboratory parameters, diabetic neuropathy, early diagnosis, risk stratification

## Abstract

Diabetic neuropathy affects approximately 50% of patients with diabetes, causing significant health issues and reducing quality of life. This review examined the associations between diabetic neuropathy and various laboratory parameters, including metabolic indicators, renal and hepatic function parameters, inflammatory biomarkers, thyroid hormones and vitamins. By summarizing the latest evidence on these easily accessible clinical parameters, the article aims to improve risk stratification, enable earlier diagnosis and develop personalized therapeutic strategies, emphasizing the potential of multi-parameter biomarker integration to optimize patient care.

## 1 Introduction

The prevalence of diabetes mellitus(DM)has kept rising globally. According to the International Diabetes Federation (IDF )Diabetes Atlas, 11th edition (2025), approximately 589 million adults aged 20–79 years had diabetes in 2024, accounting for 11.1% of the global population in this age group (1). Diabetic neuropathy, one of the most prevalent chronic complications of diabetes, DPN affects approximately 20% of both type 1 and type 2 diabetes patients within 20 years of diagnosis (2), mainly includes diabetic peripheral neuropathy (DPN), autonomic neuropathy, diabetic central neuropathy, diabetic neuropathic pain, and diabetic motor neuropathy, all characterized by nerve damage resulting from chronic metabolic disturbances induced by hyperglycemia (3). Underlying pathophysiological mechanisms include hyperglycemia-driven oxidative stress, advanced glycation end-product accumulation, microvascular dysfunction and chronic low-grade inflammation (4, 5). These mechanisms ultimately lead to progressive axonal degeneration and demyelination.

Despite its high prevalence, diabetic neuropathy often remains asymptomatic during the early stages and remains undetected until irreversible nerve damage has occurred. This diagnostic limitation underscores the critical need for serum biomarkers that can reflect early pathophysiological alterations. Such biomarkers would enable noninvasive risk stratification, preclinical detection, and timely intervention to prevent permanent neurological disability.

Accumulating evidence suggests that routine clinical biochemical parameters may serve as accessible indicators of neuropathy risk and potential determinants of its pathogenesis (6). This review systematically evaluates the current literature on the associations between these easily accessible laboratory markers and diabetic neuropathy. The aim is to facilitate the early detection of neuropathy and the development of targeted intervention strategies, even in asymptomatic diabetic populations.

## 2 Literature search strategy

We conducted a comprehensive literature search in PubMed for English-language articles published between January 2019 and November 2025. Search terms for neuropathy included: (“diabetic neuropathy” OR “diabetic peripheral neuropathy” OR “painful diabetic neuropathy” OR “diabetic autonomic neuropathy” OR “diabetic polyneuropathy”). These were combined with terms for laboratory parameters: (“human” OR “patient”) AND (“glucose” OR “C-peptide” OR “insulin resistance” OR “liver function” OR “renal function” OR “lipid profile” OR “uric acid” OR “inflammatory marker” OR “thyroid” OR “vitamin”) AND (“risk factor” OR “association” OR “correlation” OR “predictor”).

## 3 Glucose and insulin-related parameters with diabetic neuropathy

Glycemic variability and insulin resistance drive peripheral nerve injury through metabolic stress and microvascular dysfunction. Accumulating evidence suggests that markers of glycemic control and insulin sensitivity are independently correlated with the risk and progression of diabetic neuropathy, thereby highlights the importance of precise metabolic control in neuropathy prevention.

### 3.1 HbA1c

As the gold standard for evaluating long-term glycemic control, HbA1c offers a reliable measure of average blood glucose levels over 2–3 months. Accumulating evidence has indicated that elevated HbA1c levels (above the normal range) are associated with both the onset and progression of diabetic neuropathy (7–10). Zhang et al. (11) also have found a positive correlation between elevated HbA1c levels and the severity of DPN. In a retrospective cohort study involving 1,091pariticipants, Cheng et al. (12) further found that an HbA1c level of ≥7.7% could serve as a predictive threshold for neuropathic pain, and patients with HbA1c ≥7.7% exhibited a 3.15-fold higher risk of developing DPN compared to those with lower HbA1c levels(**Table 1**). In addition, evidence suggests that maintaining HbA1c within the range of 6.5%-7.0% may be optimal for preventing the progression of DPN in patients with T2DM (13) (**Table 1**).

**Table 1:** Associations between circulating glucose and insulin-related parameters and diabetic neuropathy risk.

Parameter	Increment / cut-off	Neuropathy	Risk change	Ref.
HbA1c	Elevated (continuous ≥7.7%	DPN onset /progression DPN	Positive correlation 3.15-fold increase	(7–10) (12)
	6.5-7.0%	DPN progression	Optimal control range	(13)
FCP 2h-CP	Lower levels Higher FCP/DD ratio	DPN DPN	Increased risk Protective (independent protection)	(15) (18)
	Higher levels	DPN	Independent predictor	(19)
C_2_/C_0_	Higher ratio	DPN	Protective factor	(20)
C-peptide	1.2-2.8ng/mL	PDPN	U-shaped: Elevated risk	(21)
	< 0.8 or > 3.5 ng/mL ≥1,550 pmol/L	PDPN Possible DPN	U-shaped: Protective aPR 1.72 (positive correlation)	(21) (22)
HOMA2-IR	> 1.735	CAN	Higher risk threshold	(23)

HbA1c, glycated hemoglobin; FCP/DD, Fasting C-peptide/diabetes duration ratio; 2h-CP, 2hour postprandial C-peptide.

C_2_/C_0_ = postprandial 2hour C-peptide to fasting C-peptide ratio; PDPN, painful diabetic peripheral neuropathy; HOMA-IR, The Homeostasis Model Assessment of Insulin Resistance; aPR, adjusted Prevalence Ratio; CAN, cardiac autonomic neuropathy.

### 3.2 C-peptide

C-peptide, a polypeptide co-secreted with insulin by pancreatic β-cells, serves as a critical biomarker for evaluating β-cell secretory function. Beyond its role in glucose homeostasis, accumulating evidence has found the pleiotropic effects of C-peptide, including anti-inflammatory, immunomodulation, and neuroprotective properties, which are increasingly recognized as playing a crucial role in modulating diabetic complications (14).

Clinical studies have consistently indicated significantly lower serum C-peptide levels in patients with DPN compared to non-DPN controls (15), and fasting C-peptide levels were found to be negatively correlated with the incidence DPN (16–18). In a cross-sectional study of 816 T2DM patients, Fu et al. (18) found higher ratio of fasting C-peptide to diabetes duration ratio (FCP/DD) ratio provided independently protection against DPN, exhibiting superior value to either FCP or diabetes duration (**Table 1**), suggesting the potential utility of FCP/DD as a composite predictive biomarker for DPN. Similarly, Cao et al. (19) found that 2-hour postprandial C-peptide (2h-CP) could be an independent predictor of DPN (**Table 1**). In a retrospective cohort study involving 1,278 patients with diabetes, Lian et al. (20) applied machine learning (XGBoost) to develop a DPN prediction model and identified the postprandial-to-fasting C-peptide ratio (C2/C0) as a protective factor for DPN through SHAP analysis (**Table 1**). Therefore, circulating C-peptide levels or their derived ratios might serve as potential DPN predictors.

However, evidence regarding the association between C-peptide levels and painful diabetic peripheral neuropathy (PDPN) remains inconsistent and controversial. Li et al. (21) reported a U-shaped relationship between serum C-peptide concentrations and PDPN risk: the prevalence of PDPN was elevated when serum C-peptide levels ranged from 1.2 to 2.8 ng/mL, whereas concentrations below 0.8 ng/mL or above 3.5 ng/mL appeared protective, which correlated with a reduced risk of PDPN (**Table 1**). In contrast, in multicenter cross-sectional study conducted by Christensen et al. (22) found a linear positive correlation between C-peptide and DPN, but no statistically significant association was observed between C-peptide concentrations and PDPN (**Table 1**). These conflicting results may be due to the differences in study design, patient characteristics and diagnostic criteria for PDPN. Furthermore, the potential U-shaped relationship between C-peptide and neuropathy risk suggests that conventional linear statistical models may be insufficient to capture its complex biological effects.

### 3.3 Insulin resistance index

The Homeostasis Model Assessment of Insulin Resistance (HOMA-IR) is a well-validated parameter for peripheral insulin sensitivity derived from fasting glucose and insulin concentrations. Elevated HOMA-IR reflects impaired tissue insulin sensitivity when elevated, which is a key pathophysiological mechanism of microvascular complications. Both HOMA-IR and HOMA2-IR are used to assess insulin resistance, but HOMA2-IR is considered to be the more accurate than HOMA-IR of the two. Using the updated HOMA2-IR algorithm, Liu et al. (23) found that patients with diabetic cardiac autonomic neuropathy (CAN) exhibited significantly increased insulin resistance in compared to non-CAN controls., and the severity of autonomic dysfunction was positively correlated with HOMA2-IR values and the HOMA2-IR threshold of >1.735 was associated with higher risk of CAN(**Table 1**). This suggests its potential role in the clinical risk stratification of patients with DM and targeted therapeutic intervention in patients at high risk of CAN.

Therefore, the integration of HbA1c levels, C-peptide-derived indices, and HOMA2-IR into routine assessments would enable the early risk stratification of DPN and personalised intervention. Future research should clarify the contradictory role of C-peptide in PDPN and validate composite prediction models combining these metabolic markers.

## 4 Correlations of hepatic, renal function and metabolic parameters with diabetic neuropathy

Hepatic enzymes, renal filtration markers, lipid profiles, and uric acid levels play critical roles in modulating the progression and clinical phenotype of diabetic neuropathy through metabolic detoxification and neurotoxic substance accumulation. Accumulating evidence has indicated that these organ-specific and metabolic parameters frequently coexist with diabetes and independently exacerbate nerve injury beyond glycemic control (3) which underscores their importance in comprehensive DPN risk assessment.

### 4.1 Hepatic parameters

Emerging evidence has indicated the potential role of hepatic dysfunction in the pathogenesis of diabetic neuropathy. Multiple serum hepatic parameters have been shown to exhibit significant correlations with diabetic neuropathy, including alanine aminotransferase (ALT), aspartate aminotransferase (AST), and albumin (ALB) (Figure). Yan et al. (24) found that the AST/ALT ratio (ARR) was significantly elevated in T2DM patients with DPN in comparison to those without DPN (Figure), suggesting ARR has the potential to serve as a novel and promising biomarker for the risk stratification of DPN in T2DM patients.

Serum ALB, a key marker of hepatic synthetic function, has been found to exhibit a significant inverse correlation with the prevalence and severity of DPN (25, 26) (Figure). Lower serum ALB levels have been associated with reduced nerve conduction velocity and increased severity of DPN (25), and hypoalbuminemia (serum ALB <35 g/L) has been associated with approximately 2.8-fold increased risk of DPN compared to normal levels (≥35 g/L) (25). Additionally, patients in the lowest quartile of serum ALB exhibited significantly lower nerve conduction velocity and higher vibration perception thresholds compared to those in the highest quartile (26), suggesting that maintenance of adequate serum albumin levels may represent a potential therapeutic target for neuropathy prevention in diabetic populations.

Evidence has indicated that both the presence of hepatic steatosis and the severity of hepatic fibrosis are associated with diabetic neuropathy in patients. As posited by Huang et al. (27) found that among 520 T2DM patients, DPN prevalence was significantly higher in those with hepatic steatosis and fibrosis, with liver fibrosis remaining independently associated with DPN after multivariate adjustment(Figure). Similarly, Kim et al. (28) evaluated the predictive value of non-invasive hepatic fibrosis markers in a cohort of T2DM subjects, and found that a FIB-4 index≥1.3 could serve as an independent risk predictor for DPN. Silibinin, an adjuvant therapeutic agent for liver diseases, has been shown to alleviate the symptoms of DPN largely by reducing oxidative stress and exerting anti-inflammatory effects (29).

### 4.2 Renal function

Previous studies have established a close relationship between renal dysfunction and DPN. Lai et al. (30) has the investigated the relationship between the urinary albumin-to-creatinine ratio (UACR) and the risk of developing DPN, and found that UACR≥98.6 mg/g was a critical threshold for increased DPN risk (Figure). In a 6-year prospective study, Wang et al. (31) found that in T2DM patients, lower baseline eGFR was independently associated with the incident DPN, whereas elevated serum Cr predicted DPN development exclusively in patients aged <65 years, indicating age-dependent predictive values of renal function markers for DPN. Notably, Zhang et al. (32) found that, in a cohort of 221 DPN patients with preserved renal function (eGFR ≥60 mL/min/1.73m²), eGFR was positively correlated with DPN risk in T2DM patients (Figure). This counterintuitive association may reflect a neurohemodynamic compensatory regulatory mechanism activated during the early renal hyperfiltration phase, a characteristic feature of diabetic kidney disease (DKD) even in patients with seemingly normal eGFR. Given the intricate relationship between renal function and DPN, conducting subgroup analyses stratified by distinct renal function stages is imperative to elucidate these associations.

### 4.3 Serum lipids

In a meta-analysis encompassing 39 studies, Cai et al. (33) has found that, patients with DPN had significantly higher serum triglyceride (TG) and low-density lipoprotein cholesterol (LDL-C) levels, as well as lower high-density lipoprotein cholesterol (HDL-C) levels, compared to diabetic patients without DPN(Figure). However, inconsistent evidence found by Chang et al. (34) has indicated that, DPN patients showed significantly lower serum total cholesterol (TC) and LDL-C levels compared to non-DPN controls (Figure). This paradoxical association may be attributable to impaired cholesterol availability for axonal regeneration following diabetic nerve injury. Excessively low cholesterol levels may impede the reparative processes necessary for damaged nerves. As Jende et al. (35) also found that, severely reduced serum TC levels were not only positively correlated with neuropathy severity but also associated with a progressive decline in both nerve conduction velocity and action potential amplitude. The discrepancies in conclusions across studies are likely attributable to two factors: the stage of the disease and variations in measurement. The presence of atherogenic dyslipidaemia has been observed in patients with early-stage DPN. By contrast, the advanced stage exhibit altered lipid profiles, partly because less cholesterol is required for axonal repair. Moreover, discrepancies in the specific lipid parameters measured in different studies have been associated with inconsistent results. The relationship between lipid profiles and DPN remains incompletely elucidated and warrants further investigation.

### 4.4 Serum uric acid

Recent studies regarding the correlation between dysregulated UA metabolism and DPN remain controversial. It has been established that UA exerts a concentration-dependent dual effect: at physiological concentrations, it functions as an antioxidant by scavenging free radicals and protecting neuronal cells, whereas at high concentrations, it acts as a pro-oxidant, promoting damage to both microvascular and neural cells (36).

Wang et al. (16) found that elevated serum UA levels within the normal physiological range were associated with a reduced risk of DPN. In a case-control study, Zhuang et al. (37) observed that DPN patients had significantly lower serum UA levels than non-DPN patients, with a serum UA cutoff value of 324 μmol/L, below which the risk of DPN was found to increase(Figure). As Zhang et al. (38) have also found that, lower serum UA is an independent risk factor for DPN in T2DM patients, and decreased UA levels may specifically influence the motor conduction velocity of the tibial nerve.

**Figure 1:**
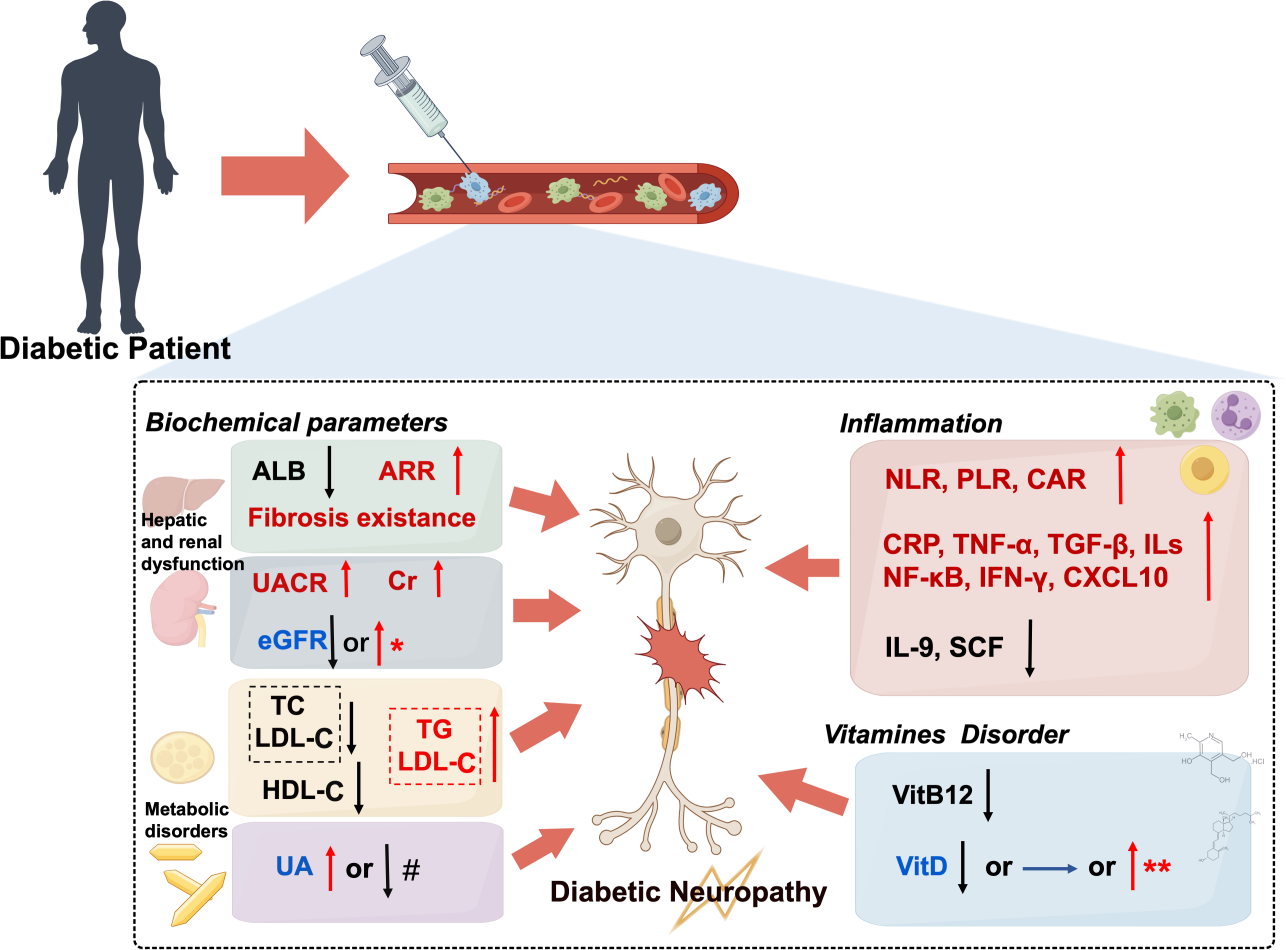
Clinical parameters associated with diabetic neuropathy: consistent versus inconsistent evidence. Multiple clinical parameters are consistently associated with diabetic neuropathy, including hepatic dysfunction (elevated ARR, decreased ALB, and fibrosis existence), renal impairment (elevated UACR and Cr), dyslipidemia, chronic inflammation (elevated NLR, PLR, CAR, CRP, TNF-α, TGF-β, ILs, NF-κB, IFN-γ, and CXCL10; decreased IL-9 and SCF), and vitamin B12 deficiency. Conversely, associations between diabetic neuropathy and estimated eGFR, UA, and vitD remain inconsistent: both decreased and increased eGFR (* in patients with preserved renal function), elevated and decreased UA (# UA<324 μmol/L), and reduced, unchanged, or paradoxically elevated vitD (** in vitD insufficient group) have been found correlated with diabetic neuropathy. ALB, albumin; ARR, aspartate aminotransferase/alanine aminotransferase ratio; CAR, C-reactive protein-to-albumin ratio; Cr, creatinine; CXCL10, C-X-C motif chemokine ligand 10; eGFR, estimated glomerular filtration rate; HDL-C, high-density lipoprotein cholesterol; IFN-γ, interferon-γ; IL-9, interleukin-9; LDL-C, low-density lipoprotein cholesterol; NLR, neutrophil-to-lymphocyte ratio; PLR, platelet-to-lymphocyte ratio; SCF, stem cell factor; TC, total cholesterol; TG, triglycerides; UA, uric acid; UACR, urinary albumin-to-creatinine ratio; VitB12,vitamin B12; vitD, vitamin D. This Figure was created by Figdraw.

Conversely, data have shown the positive associations between elevated serum UA and increased DPN risk (Figure). A multicenter study conducted in Iran found that, patients with DPN had significantly higher serum UA levels than patients without DPN. The risk of DPN increased progressively as serum UA levels increased (39). A meta-analysis encompassing 20 studies (5 cohort and 15 cross-sectional) also confirmed a positive association between elevated serum UA levels and increased DPN risk in patients with T2DM (36). Furthermore, both Kaewput et al. (40) and Zhang et al. (36) reported that elevated serum UA remained an independent predictor of DPN even after adjusting for traditional risk factors. As demonstrated by Zhang et al. (41), a dose-response effect was established, indicating that a each 1 mg/dL (approximately 59.48 μmol/L) increase in serum UA level was associated with a 17% higher risk of developing DPN. The underlying mechanism through which UA exerts its effects on DPN has been investigated in db/db diabetic mouse models, which revealed that UA exacerbates diabetic neuropathy by augmenting oxidative stress, and Xanthine oxidase inhibitors, pharmacological agents that reduce serum UA levels, could alleviate delayed nerve conduction velocity and improve intraepidermal nerve fiber density (42). (**Figure 1**).

The inconsistent outcomes of these studies are likely attributable to the non-linear dose-response relationship of UA, which functions as an antioxidant at physiological concentrations but as a pro-oxidant when elevated. Discrepancies in methodological approaches across studies, including variations in study design, ethnic background, and the adjustment for confounders such as renal function, further contribute to the heterogeneity observed.

The accessibility of metabolic biomarkers (hepatic and renal function, lipids, and uric acid) facilitates practical DPN risk stratification. The application of an integrated panel approach has the potential to facilitate more effective identification of high-risk patients than the use of isolated markers. It is, therefore, recommended that future research should establish optimal thresholds and determine whether targeted metabolic interventions can effectively delay the onset of DPN.

## 5 Inflammatory markers and diabetic neuropathy

Emerging evidence indicates that chronic low-grade inflammation serves as a critical pathological mediator between sustained hyperglycemia and DPN (43). This inflammatory response contributes to both demyelination and axonal injury. Routine hematologic inflammatory indices and circulating cytokine levels not only reflect disease activity but also represent accessible biomarkers for DPN risk assessment.

### 5.1 Inflammation parameters derived from routine blood tests

Emerging evidence suggests a correlation between elevated levels of multiple inflammatory parameters derived from routine hematology test and the development of microvascular and macrovascular complications of diabetes. These inflammatory parameters include the neutrophil-to-lymphocyte ratio (NLR), platelet-to-lymphocyte ratio (PLR), and the C-reactive protein-to-albumin ratio (CAR). For instance, in a meta-analysis of 16 studies, Shahrabi et al. (44) found that patients with DPN had significantly higher NLR than those without DPN. In a prospective study, Chen et al. (45) found that elevated NLR and PLR were associated with DPN, with PLR exhibited higher diagnostic value in T1DM and NLR in T2DM. Elevated baseline NLR also predicts future DPN development in T1DM patients (Figure). Similarly, Aktas (46) found that the CAR in T2DM patients with DPN was significantly higher than those without neuropathy (Figure), and high CAR was found to be independently associated with the increased risk of neuropathy and might function as a potential biomarker for DPN in the T2DM population (**Figure 1**).

### 5.2 Peripheral blood inflammatory cytokines

Studies have explored the associations between peripheral inflammatory cytokines and the risk of DPN (47, 48). Ascaso et al. (49) and Abdulrhaman et al. (50) both found that levels of inflammatory cytokines (e.g., chemokine (C-X-C motif) ligand 10 (CXCL10), tumor necrosis factor-alpha (TNF-α) and transforming growth factor-beta (TGF-β) were significantly higher in patients with DPN compared to patients without DPN (Figure). Furthermore, numerous investigations have demonstrated a positive correlation between elevated levels of various inflammatory markers and increased DPN susceptibility, such as C-reactive protein (CRP), interleukins (ILs), TNF-α, nuclear factor-kappa B (NF-κB), interferon-gamma (IFN-γ), and CXCL10 (47, 48) (Figure). Nevertheless, some inflammatory factors, such as interleukin-9 (IL-9) and stem cell factor (SCF), have been shown to exert protective effects against DPN (47), suggesting the presence of complex regulatory inflammatory pathways in DPN (Figure).

Thus, circulating inflammatory markers may enhance the risk stratification of DPN and represent a novel target for therapeutic intervention.

## 6 Thyroid hormones and diabetic neuropathy

Thyroid hormones play a pivotal role in regulating key neurodevelopmental and neuroprotective processes, including the growth and repair of nerve fibers and the modulation of neuronal cellular function (51) Accumulating evidence has indicated the correlation between thyroid dysfunction and DM (52–55), as well as the development of diabetic complications (56).

### 6.1 Hyperthyroidism and subclinical hyperthyroidism

Previous studies conducted by Patel et al. (56) and Mehalingam et al. (57) have consistently found no significant association between overt hyperthyroidism and DPN, nor with other diabetic complications (**Table 2**). Notably, the specific association between subclinical hyperthyroidism and diabetic neuropathy remains largely unexplored, as original investigations addressing this clinical question are virtually absent (**Table 2**).

**Table 2:** The relationship between thyroid hormones and diabetic neuropathy.

Thyroids hormones	Subjects	The relationship with diabetic neuropathy	Ref.
Hyperthyroidism			
Clinical	T2DM patients	No significant association	(56, 57)
Subclinical	–	Largely unexplored	--
Hypothyroidism			
Clinical	T2DM patients	Positive correlation; independent risk factor	(56)
Subclinical	T2DM patients	Independent predictor of DPN severity	(58)
Euthyrodism			
FT3: low-normal FT3: normal	T2DM patients T2DM patients	Inverse correlation No association	(60, 62) (63)
FT4: low-normal FT4: high-normal	T2DM patients T2DM patients	Inverse correlation Positive correlation	(60, 63, 64) (61)
TSH	T2DM patients T2DM patients	Positive correlation Weak inverse correlation	(60) (63)

FT4, Free Thyroxine; FT3, Free Triiodothyronine; TSH, Thyroid-Stimulating Hormone; DPN, Diabetic peripheral neuropathy; T2DM, type 2 diabetes mellitus; SCH, subclinical hypothyroidism; SCH mice, SCH-induced mice.

### 6.2 Hypothyroidism and subclinical hypothyroidism

Growing evidence has identified a correlation between clinical and subclinical hypothyroidism (SCH) and diabetic complications, including DPN. In a cross-sectional study, Patel et al. (56) found that hypothyroidism was positively associated with DPN and was an independent risk factor for DPN (**Table 2**). As Allam et al. (58) found, patients with DPN who also had SCH exhibited more severe neuropathy symptoms compared to those without SCH. Furthermore, SCH was found to be an independent predictor of DPN severity (**Table 2**). Fan et al. (59) investigated the potential mechanisms underlying this association, in which T2DM mice with SCH developed more severe peripheral neuropathy compared to diabetic mice without SCH. This exacerbation was attributed to palmitoylation-induced modifications of the thyroid-stimulating hormone receptor (TSHR) on Schwann cell surfaces, which consequently triggered increased reactive oxygen species (ROS) generation and mitochondrial dysfunction, contributing to accelerated peripheral nerve damage in DPN.

### 6.3 Euthyroidism and DPN

Free Triiodothyronine (FT3): Studies have indicated an inverse correlation between serum FT3 levels and the prevalence of DPN in T2DM patients with euthyroidism (60–62) (**Table 2**), suggesting that lower FT3 levels, even when within the normal reference range, may contribute to the development of peripheral nerve dysfunction. However, Sun et al. (63) found no significant association between FT3 and DPN (**Table 2**). Variations in study population might contribute to these inconsistent findings.

Free Thyroxine (FT4): The evidence regarding the association of FT4 with DPN in euthyroid T2DM patients is also inconsistent. Several studies have found the correlations between lower FT4 levels within the normal range and increased risk of DPN (60, 63, 64) (**Table 2**). As demonstrated by Lin et al. (61), a positive correlation was found between FT4 and DPN prevalence(**Table 2**). Whether a U-shaped relationship exists between FT4 levels and DPN risk remains unclear and requires further investigation.

Thyroid-Stimulating Hormone (TSH): conclusions regarding TSH and DPN in euthyroid T2DM patients are also conflicting. In a retrospective analysis of 248 euthyroid patients with T2DM, Hu et al. (60) described a positive correlation between TSH levels and DPN (**Table 2**). However, Sun et al. (63) found a weak inverse correlation between TSH and DPN after adjusting for potential confounding variables (**Table 2**).

Notable inconsistencies in the associations between thyroid parameters and DPN among euthyroid T2DM patients likely reflect heterogeneity in study populations, methodological variations, and disparate confounder adjustment strategies. Although routine thyroid screening represents a cost-effective approach for DPN risk stratification, particularly given established links between subclinical hypothyroidism and neuropathy progression, conflicting data regarding euthyroid hormone levels underscore the need for standardized, large-scale prospective studies to clarify causal relationships and facilitate integration of thyroid status into personalized risk prediction models.

## 7 Vitamins and diabetic neuropathy

Vitamin D and vitamin B12 play essential roles in maintaining peripheral nerve integrity through neurotrophic support, myelination maintenance, and immunomodulation. Accumulating evidence has indicated significant associations between deficiencies in these micronutrients and the development or progression of DPN.

### 7.1 Vitamin B12

Vitamin B12 (cobalamin) plays a pivotal role in maintaining the integrity of the nervous system, primarily by supporting myelin synthesis and facilitating nerve regeneration (65). Vitamin B12 deficiency has been well-documented as a cause of both motor and painful neuropathies, irrespective of other etiologies (66). Clinical evidence has indicated a strong association between vitamin B12 deficiency and DPN (Figure). As indicated by the findings of Jin et al. (67), there is a bidirectional association between vitamin B12 deficiency and DPN, with B12 deficiency significantly increasing DPN risk and conversely, DPN patients exhibiting a higher incidence of B12 deficiency. In a longitudinal cohort study, Bell (68) found that long-term metformin use frequently leads to vitamin B12 deficiency, which may subsequently contribute to the development or exacerbation of distal symmetrical polyneuropathy and autonomic neuropathy in diabetic patients.

The administration of vitamin B12 supplementation has been shown to have therapeutic potential in the treatment of DPN. In a 12-month intervention study, Didangelos et al. (69) found that patients who received oral mecobalamin (a bioavailable form of vitamin B12) showed significant improvements across multiple clinical and neurophysiological outcomes relative to untreated controls, including increased serum vitamin B12 levels, enhanced neurophysiological parameters, improved sweating function, reduced pain scores and improved quality of life (**Figure 1**).

### 7.2 Vitamin D

Vitamin D plays crucial role in DM and its complications, with deficiency consistently implicated in the onset and progression of these conditions (70, 71). Beyond its metabolic effects, vitamin D exerts neuroprotective effects mediated by its receptors, which are widely expressed in neurons, oligodendrocytes, and glial cells—key cell types involved in peripheral nerve function and repair (72).

The correlations between vitamin D levels with diabetic neuropathy has been widely discussed in recent years. Clinical studies have shown that patients with diabetic neuropathy exhibit lower levels of vitamin D compared to those without diabetic neuropathy (73, 74). Moreover, vitamin D deficiency has been identified as an independent risk factor for DPN (75) and PDPN (76, 77) (Figure). In elderly patients with T2DM, Fei et al. (75) found that vitamin D deficiency may promote development of DPN by prolonging the neural motor latency in large-diameter nerve fibers. Notably, however, it had no significant impact on small-fiber nerve function. A prospective study (78) indicated that elevated serum 25-hydroxyvitamin D(25- (OH) D) levels were associated with a reduced risk of diabetic microvascular complications, including neuropathy. However, a Mendelian randomization analysis by Huang et al. (79) failed to detect a causal relationship between serum 25- (OH) D levels and diabetic neuropathy in the Chinese population (Figure). Despite this, administration of vitamin D supplement has been found to improve neuropathic pain in patients with DM, suggesting a potential for vitamin D supplements to promote neurological recovery, and impede the progression of nerve damage (80–83).

In a large cross-sectional study of 4,435 T2DM patients, Yang et al. (84) observed an inverse association between serum 25-(OH)D levels and neuropathy symptoms(Figure). However, this relationship was dependent on vitamin D status: within the vitamin D insufficiency group, patients with symptomatic DPN paradoxically had significantly higher serum 25(OH)D levels than asymptomatic patients, whereas within the vitamin D sufficiency group, symptomatic individuals had significantly lower 25(OH)D levels. Therefore, the association between serum 25(OH)D and neuropathy symptoms is influenced by an individual’s baseline vitamin D status (**Figure 1**).

These findings highlight the clinical utility of routine screening and correction of vitamin B12 and D deficiencies as strategies to attenuate DPN progression. Nevertheless, the implementation of randomized controlled trials is warranted to establish causal relationships, optimize supplementation protocols, and elucidate potential synergistic benefits of combined vitamin administration.

## 8 Limitations

Several limitations should be acknowledged. Firstly, due to the nature of narrative reviews, we did not conduct a formal quality assessment or hierarchical grading of the evidence for the included studies. The primary objective of this review was to provide a comprehensive overview and conceptual synthesis of existing literature rather than to generate quantitative effect estimates or graded clinical recommendations. Consequently, the substantial heterogeneity in study designs, ranging from randomized controlled trials and Mendelian randomization studies to cross-sectional observations and preclinical investigations, precluded standardized evidence grading across all included articles. The presented findings should be interpreted with caution, given that the strength of causal inference varies considerably across different biomarkers and clinical parameters. Future systematic reviews with formal risk-of-bias assessment and, where appropriate, meta-analyses are warranted to establish definitive evidence levels and clinical guidelines.

## 9 Conclusion and perspective

Together, routine laboratory parameters, including glucose-metabolic indices, inflammatory markers, hepatic and renal function, and micronutrients, offer significant additional value for diabetic neuropathy risk stratification beyond traditional glycemic assessment. Integrating these multidimensional biomarkers into composite predictive models is a significant improvement over single-marker approaches because these models can identify high-risk subgroups that require intensive intervention. Future research should include rigorous prospective cohort studies to validate population-specific thresholds, machine learning algorithms to optimize multi-parameter risk scoring, and randomized interventional trials to establish causality between biomarker correction and neuropathy trajectory modification. These studies are necessary for translating these laboratory findings into actionable clinical protocols.
